# *Shilajit:* A panacea for high-altitude problems

**DOI:** 10.4103/0974-7788.59942

**Published:** 2010

**Authors:** Harsahay Meena, H. K. Pandey, M. C. Arya, Zakwan Ahmed

**Affiliations:** *Herbal Medicine Division, Defence Research and Development Organization, DIBER, Field Station, Pithoragarh - 262 501, Uttarakhand, India*

**Keywords:** Fulvic acid, herbomineral drug, high-altitude problems, hypoxia, *Shilajit*, mineral pitch, rejuvenator

## Abstract

High altitude problems like hypoxia, acute mountain sickness, high altitude cerebral edema, pulmonary edema, insomnia, tiredness, lethargy, lack of appetite, body pain, dementia, and depression may occur when a person or a soldier residing in a lower altitude ascends to high-altitude areas. These problems arise due to low atmospheric pressure, severe cold, high intensity of solar radiation, high wind velocity, and very high fluctuation of day and night temperatures in these regions. These problems may escalate rapidly and may sometimes become life-threatening. *Shilajit* is a herbomineral drug which is pale-brown to blackish-brown, is composed of a gummy exudate that oozes from the rocks of the Himalayas in the summer months. It contains humus, organic plant materials, and fulvic acid as the main carrier molecules. It actively takes part in the transportation of nutrients into deep tissues and helps to overcome tiredness, lethargy, and chronic fatigue. *Shilajit* improves the ability to handle high altitudinal stresses and stimulates the immune system. Thus, *Shilajit* can be given as a supplement to people ascending to high-altitude areas so that it can act as a “health rejuvenator” and help to overcome high-altitude related problems.

## INTRODUCTION

Various environmental and physical factors adversely affect physiological functions of the body at high altitudes (more than 2500 meters above sea level).[1] These factors are low humidity, low atmospheric pressure, severe cold, high wind velocity, and high intensity of solar radiation.[2–4] The common problems associated with soldiers or people going to high altitudes are acute mountain sickness (AMS), high-altitude pulmonary edema (HAPE), high-altitude cerebral edema (HACE), hypoxia, insomnia, lack of appetite, tiredness, lethargy, stomach upset, disinclination to work, and bone and muscle degradation with the result that the individual becomes physically and mentally depressed after reaching high altitudes.[5–9] These problems may escalate rapidly and the results may also be lethal sometimes.

*Shilajit*, a herbomineral drug, contains ample amounts of fulvic acid and mineral constituents.[10–13] The fulvic acid stimulates blood formation, energy production, and prevents cold exposure and hypoxia.[14–18] It actively takes part in the transportation of nutrients into deep tissues and helps to overcome tiredness, lethargy, and chronic fatigue.[14,15,17–19] It also works effectively as a tonic for cardiac, gastric, and nervous systems, adaptogen and antistress agent.[15–18,20–23] In view of these properties, *Shilajit* can be used as a supplement to overcome high-altitude-related problems.

## SHILAJIT

*Shilajit* is a Sanskrit word meaning “Conqueror of mountains and destroyer of weakness” and ‘Winner of rock’.[24] Its other names are *Silajit* and *Silaras* in Sanskrit,[15] Shilajita Mumiyo, Mineral pitch, Asphalt, Jew's pitch, Mineral wax, or Ozokerite in English. [25–28] *Shilajit* is a pale-brown to blackish-brown exudate in the form of a smooth and clean gum that comes out of the mountains in the months of May-July.[25,27,29,30] *Charaka Samhita* describes *Shilajit* as “Stones of metal like gold” while *Sushruta Samhita* describes it as a “A gelatinous substance.”[25,27] *Rasarangini and Dwarishtarang* also claim that *Shilajit* is an exudate of a latex gum-resin of plants.[10] This substance is known as *Çilájatu* and cures all distempers of the body”.[25,27] It is found in the lower Himalayan hills at altitudes between 1000 to 5000 meters in Uttarakhand, Himachal Pradesh, Kashmir, and Arunachal Pradesh.[23] It is also found in Afghanistan, Nepal, Bhutan, Pakistan, China, Tibet, and the former U.S.S.R.[11,15,23] *Shilajit* is composed of humus and organic plant materials that have been compressed by layers of rocks.[12,26,31] The humus consists of 60-80% organic matter, is bitter in taste, and has an odor like cow's urine.[11,12,18] It contains dibenzo-alphapyrones and related metabolites, small peptides, humic acid, some lipids, uronic acids, phenolic glucosides, amino acids, and fulvic acid.[10–13,32,33] It also contains more than 84 minerals including copper, silver, zinc, iron, and lead in their ionic forms.[12,13,18] *Shilajit* is processed by several drug manufactures and marketed in capsule form for human consumption.[18,34,35] It is a natural medicine with a long history of human use for healing and performance-enhancement in diabetes and the urinary, immune, digestive, cardiac, and nervous systems.[13,15–18,21,22,25,26,31,34,35] Thus, *Shilajit* is an ayurvedic medicine with many properties and is capable of treating almost all kinds of body ailments.[25–27,31,36,37]

## HYPOXIA AND WEAKNESS INCLUDING MUSCULAR DEGRADATION

*Shilajit* amplifies the benefits of other herbs by enhancing their bioavailability in the body.[16] It contains more than 84 types of minerals, including the main ingredient, fulvic acid,[12,13,18] and provides most of the essential minerals to the body.[13,16] Fulvic acid acts as a carrier molecule in the human system, helps in the transportation of nutrients into the deep tissues, and removes deep-seated toxins from the body.[19] *Shilajit* helps in energy production, reduces the recovery period of injured muscles, bones, and nervous system,[14,15,18] and is used in the treatment of fractures. [13,15,18] Hence, it reduces the degradation of muscles and bones, and increases their strength at high altitudes. *Shilajit* also has the ability to overcome physical as well as mental stress[17,18,34] due to which it is used in the treatment of tiredness, lethargy, cold etc. which are common problems at high altitudes. Bucci (2000)[14] and Frawley *et al*., (2001)[17] have reported that the consumption of *Shilajit* with milk constitutes one of the strongest natural supplements with the potential to eliminate all kinds of weakness. Fulvic acid helps in the absorption of iron into the body, making it bioavailable to bone marrow stem cells for blood formation[16,25] and hence, *Shilajit* can be very helpful in coping with hypoxia-like conditions in the body. Apart from its blood-purifying property, *Shilajit* also enhances the oxygen-carrying capacity of the blood. [16,25] It helps in improving blood circulation and its diffusion into tissues, and maintains the necessary oxygen levels in the body during hypoxia.[17] Dash (1991)[16] has reported that *Shilajit* is a good detoxifier, helping in the elimination of toxins from the body, and its regular use stops the production of toxins in the body. Hence, *Shilajit* is also used in the treatment of carbon dioxide poisoning in hilly areas.

## ACUTE MOUNTAIN SICKNESS (AMS)

The major problem associated with high altitudes is acute mountain sickness (AMS). The symptoms of AMS are gastrointestinal distress, constipation, diarrhea, nausea, vomiting, headache, anorexia, breathlessness, dry nose, tiredness, giddiness, palpitation, cough, lethargy, dyspnea, edema, dizziness, fatigue, disturbed sleep, and disinclination to work.[1,38] Various scientists have experimentally proved that *Shilajit* can be used effectively for the treatment of gastrointestinal distress,[15,16] headache, weakness[17,18,25,34] anorexia, heart problems,[16] dehydration, insomnia, dyspnea,[15,16] moist cough,[39] and pain.[40] Hence, it can be very beneficial for the treatment of AMS. Insomnia is another common problem of high altitudes—it develops due to hypoxia, stress, anxiety among other causes. *Shilajit* acts as an antistress,[18,34,41] antianxiety,[23] antiepileptic,[17] and adaptogenic agent[18,42] and has also been found to be useful in the treatment of the insomnia.

## HIGH-ALTITUDE PULMONARY EDEMA AND PAIN (HAPE)

HAPE is a major problem at high altitudes, which develops due to oxygen deficiency as well as low atmospheric pressure.[43–45] In HAPE, fluids are accumulated in the lungs of the body and some of the symptoms are shortness of breath, chest pain, fever, lethargy, cough, and cyanosis.[1] Being a diuretic agent, *Shilajit* removes the excess fluid from the lungs as well as the body,[17,34] which makes it very effective in the treatment of HAPE-like conditions and edema.[17] Shilajit has analgesic and anti-inflammatory effects and is thus useful for different painful conditions of the body.[24,40,46] *Shilajit* is also found to be very beneficial in rheumatoid arthritis, osteoarthritis, and gout[13,22,40] as it nourishes the joints and reduces the inflammation and pain.[13,22,47] Dash (1991)[16] has also reported that *Shilajit* can be useful in the treatment of dyspnea.

## HIGH-ALTITUDE CEREBRAL EDEMA AND DEMENTIA (HACE)

HACE is the result of swelling in the brain tissue due to low atmospheric pressure. Symptoms include headache, loss of coordination, weakness, and decreased level of consciousness, including disorientation, loss of memory, hallucination, psychotic behavior, and coma.[48,49] It generally occurs after staying for a week or more at high altitudes. Severe instances can lead to death if not treated quickly. *Shilajit* can be very useful in the treatment of HACE-like conditions of the brain as it maintains the extracellular volume in the body through its diuretic action, and removes the excess fluid and toxins from the brain as well as the body.[17,34] It improves memory and enhances confidence for better handling of stress;[13,18,23,34] it also has very good antioxidant properties.[13,16,22,47]

Dementia is one of the common problems faced by mountaineers, which occurs due to degradation of neurons, either by hypoxia or by free radicals or toxins in the brain. *Shilajit* may play a potential role in the treatment of Alzheimer's and Parkinson's diseases[24] as it is an immunostimulant and has been found to be very effective in treating immune, nervous, and urinary disorders.[13,15,16,18,21,22,34,50] Jaiswal *et al*. (1992)[23] revealed its anxiolytic activity and its usefulness in the treatment of disinclination to work. *Shilajit* is also used as a tonic in the ayurvedic system of medicine due to the presence of humic acid, fulvic acid, coumarins, and triterpenes.[17,41]

## GASTROINTESTINAL DISORDERS AND DEHYDRATION

Gastrointestinal distress (lack of appetite, constipation, diarrhea, nausea, vomiting) is another major problem seen at high altitudes[1] and dehydration is a common problem faced by mountaineers. *Shilajit* is an important drug that helps in the digestion and absorption of food in the gastrointestinal tract (GIT)[25,51] and is also useful for the treatment of nausea, vomiting, and digestive disorders.[15–17] Being an excellent source of nutrients, it used as a tonic and helps in the better utilization of food.[16,51] It stimulates the pancreas to secrete insulin and maintains an equilibrium of catabolism and anabolism in the body[13,17,25,34] It also has a laxative property in the human body.[25] Frawley (1989)[34] suggested that the *Shilajit* could also act as a gastric tonic whereas Goel *et al*., (1990)[46] revealed that *Shilajit* increases the carbohydrate/protein ratio and decreases the gastric ulcer index. The fulvic acid and 4-methoxy 6-carbomethoxybiphenyl present in *Shilajit* decrease acid-pepsin secretion, cell shedding, and also act as ulcer protectives.[40,41] Thus, *Shilajit* can be very useful to cope with common high-altitude problems like gastrointestinal disorders and dehydration.

## RADIATION PROTECTION

The high intensity of solar radiation, particularly, the high intensity of UV radiation, also affects people at high altitudes.[4] The reflection of UV and solar radiation is quite high in snowbound areas compared to the plains.[3] Hence, the risk of sunburn, skin, and eye diseases, even skin cancer, is more at high altitudes.[1] *Shilajit* can be useful for the treatment of skin and eye disorders due to its photoprotective action.[17,34]

## ACCLIMATIZATION AND IMMUNOSTIMULATION

Acclimatization is a major problem for people who go to high altitudes for the first time. *Shilajit* is very helpful in improving the immunity of the body[34,42,50] as it activates macrophages and splenocytes, and hence, has been shown to have adaptogenic properties.[42,52] Thus, it is very helpful in acclimatizing to environments in hilly areas,[50] and has also been shown to reduce tumor growth.[18,53] *Shilajit* also has anti-allergic activity against histamine releasers and has been seen to induce degranulation of mast cells.[25,52] Thus, it can also be used in the treatment of allergic conditions.

Other uses of *Shilajit* are as a lithotriptic, antiseptic,[17] anodyne, anti asthmatic agent,[22,39,47] and in the treatment of AIDS,[34] parasitic infections,[16,17] chronic fever, jaundice,[25] obesity,[25,39] sexual disorders,[17,34,54] and thyroid disorders.[55] *Astanga Hradaya* also states that it is the best rejuvenator.[39] Hence, *Shilajit* is a herbomineral formulation that acts as a panacea for altitude-related problems.

## MECHANISM OF ACTION

*Shilajit* contains active organic molecules such as fulvic acid along with minerals (in their ionic forms). The fulvic acid helps in the transportation of these minerals into cells for maintaining and restoring their electrical potency, which prevents their decay and death. *Shilajit* helps in metabolism and promotes energy production in the body. It maintains an equilibrium of catabolism and anabolism, enhances the absorptive and detoxifying capacity of the body, and stimulates the immune system and blood formation in the body.

## DOSE

The recommended dose of *Shilajit* for maintenance of optimal health is 300-500 mg/day. *Shilajit* powder taken with milk twice a day will ensure optimal blood levels and therapeutic efficacy.[27,28,34] It is slowly metabolized and reaches a maximum level in the blood after 12-14 hours of consumption.

## INDICATIONS/COUNTERACTION

*Shilajit* is safe for everyone and has been found to be quite safe for up to 3 g/kg body weight in mice.[50] It should not be used with horse gram, meat of pigeon, *Solanum nigrum* (black nightshade). [25,27,39] Raw and unprocessed *Shilajit* should not be used as it contains a significant amount of free radicals and has been found to be contaminated with different fungal organisms such as *Aspergillus niger*, *A*. *ochraceous*, and *Trichothecium roseum*.

## CONCLUSION

*Shilajit* is an ayurvedic drug with a long history of human use and has been used in nervous, diabetic, urinary, immune, cardiac, and digestive disorders, and is also used as a performance enhancer.[14–18] Traditionally, it has been recommended for the cure of almost all kinds of human diseases. Ancient works such as the Hindu Materia Medica, Charaka Samhita, and Susruta Samhita also describe the medicinal properties of *Shilajit*. Hence, it is a highly recommended drug in the *ayurvedi*c and other traditional medicine systems of India. It would certainly be helpful to fight against common high-altitude problems like hypoxia, AMS, HAPO, HACE, dehydration, UV radiation etc when taken as a supplement by people ascending to high altitudes.

In light of *Shilajit's* tremendous medicinal potential, it would not be an exaggeration to say that it can be a panacea for all human ailments and Nature's wonderful gift to mankind.
